# Blood Pressure Variations Real-Time Reflect the Conditioned Fear Learning and Memory

**DOI:** 10.1371/journal.pone.0032855

**Published:** 2012-04-04

**Authors:** Yuan-Chang Hsu, Lung Yu, Hsiun-ing Chen, Hui-Ling Lee, Yu-Min Kuo, Chauying J. Jen

**Affiliations:** 1 Institute of Basic Medical Sciences, National Cheng Kung University, Tainan, Taiwan; 2 Institute of Behavioral Medicine, National Cheng Kung University, Tainan, Taiwan; 3 Department of Physiology, National Cheng Kung University, Tainan, Taiwan; 4 Institute of Public Health, National Cheng Kung University, Tainan, Taiwan; 5 Department of Cell Biology and Anatomy, National Cheng Kung University, Tainan, Taiwan; Tokai University, Japan

## Abstract

The conditioned fear learning and memory occurs when a neutral conditioned stimulus (CS) is paired with an aversive unconditioned stimulus (US). This process is critically dependent on the amygdala and inevitably involves blood pressure (BP) alterations. We hypothesized that BP variations could instantaneously reveal individual steps during conditioned fear learning and memory. An implanted telemetric probe was used to monitor the BP real-time in rats during training and testing sessions of the fear-potentiated startle. Our results showed that (i) the conditioned fear learning during the training sessions was reflected by light (CS)-induced rapid BP elevations and by electric shock (US)-evoked sympathetic tone elevations; (ii) these two BP-related parameters were not only negatively correlated with each other but also coupled to each other in the training session trials; (iii) both parameters closely predicted the performance of fear-potentiated startle on the next day; and (iv) although local blocking of one of the two fear-conditioned pathways in the training session partially inhibited fear learning, the fear memory retrieval still used both pathways. Altogether, real-time blood pressure variations faithfully revealed the critical steps involved in conditioned fear learning and memory, and our results supported a coupling between the cued learning and the post-shock calmness.

## Introduction

Classical fear conditioning occurs when a neutral stimulus (the conditioned stimulus, CS) is paired with an aversive stimulus (unconditioned stimulus, US), such as an electric shock. After training, the previously neutral stimulus is able to elicit a variety of autonomic, hormonal, and skeletal responses that accompany the fear experience. The amygdala is an important site for associative memory storage during fear conditioning [1]–[6]. Fear-potentiated startle is an alternative measure of fear conditioning that depends on the amygdala [6]. It is widely believed that conditioned fear stimuli activate the basolateral nucleus of the amygdala (BLA) projection neurons which send afferents to two distinct target areas, i.e., the central (CeA) and the medial (MeA) nuclei of the amygdala [7], [8]. Moreover, the ventromedial nucleus of the hypothalamus (VMH), a downstream target of MeA, is involved in fear responses [9]–[13]. Interestingly, localized injection of substance P (SP) into VMH induces a typical cardiovascular defense response [14], and this neurotransmitter plays a major role in the MeA-VMH pathway when performing the task of fear-potentiated startle [15]. It is tempting to monitor blood pressure (BP)-related parameters real-time to reveal the individual steps of fear learning and memory.

In this study, we used an implanted telemetric probe to monitor real-time BP and sympathetic tone variations in conscious rats undergoing training and testing sessions of the fear-potentiated startle task. Our aims were to i) quantify the CS-induced BP changes and US-evoked sympathetic tone changes, ii) test whether and how these two parameters interact with each other, and iii) investigate whether these parameters could reflect the conditioned fear learning real-time and to predict the fear memory that was tested 1 day later. Finally, we used pharmacological approaches to locally block one of the two fear-conditioned pathways in the training session, and consequently examined whether the fear memory retrieval would use one or both pathways.

## Materials and Methods

### Animals

This animal study was conducted in conformity with the US National Institute of Health “Guide for the Care and Use of Laboratory Animals.” The animal handling procedures were approved by the Institutional Animal Care and Use Committee of National Cheng Kung University (Permit Number: 98162). All efforts were made to minimize the number of animals used and their suffering. Male Sprague-Dawley rats (7∼8-wk-old) were purchased from the National Cheng Kung University Animal Center. Animals were housed in an environmentally controlled room (temperature 23±1°C; light on at 6 AM and off at 6 PM) in groups of 5 per cage with rat chow and water *ad libitum*.

### Real-time BP Measurement and BP Spectral Analysis

BP in conscious animals was measured real-time using a biotelemetry system (Dataquest IV, Data Science International, St. Paul, MN, USA). Rats were anesthetized by brief CO_2_ exposure and followed by i.p. injection of a mixed solution (ketamine 50 mg/mL, xylazine 23.3 mg/mL, atropine 1 mg/mL; 2 mL/kg). A battery-operated telemetry transmitter (TA11PA-C40) was implanted in the abdominal cavity with its tip inserted into the abdominal aorta. The BP was continuously recorded at a sampling rate of 1000 Hz from an antenna board mounted outside of the cabinet wall. Spectral analysis was performed offline with MATLAB (Mathworks, Natick, MA, USA) on the original BP tracing to assay the sympathetic tone in conscious animals. Digital signal processing of bioelectric signals was similar to that described in our previous study [16]. Briefly, we used fast-Fourier transform along with hamming window filtering to calculate the power spectral density in time periods longer than 1 min. The spectrum was integrated between 0.27∼0.74 Hz to calculate the power distribution in low-frequency band, which served as a marker for the sympathetic activity [17].

### Behavioral Apparatus and Procedures for Fear-Potentiated Startle

Two weeks after probe implantation, rats were trained and tested in two separate but identical startle reflex systems (SR-Lab, San Diego Instruments, San Diego, CA). The startle reflex system consisted of three major units: the control unit (controlling the stimuli and monitoring the response), the startle chamber (a cylindrical enclosure equipped with steel bars to deliver electric shocks and a motion sensor to detect startle responses), and the isolation cabinet (normally dark, ventilated, and sound attenuated). The acoustic startle stimulus was a 50 ms white noise at an intensity of 95 dB. The visual CS was a 3.7 s light produced by a light bulb attached to the top of the cabinet and the US was a 0.4 mA footshock with duration of 0.5 s. In the beginning, rats were placed in a training cabinet for 10 min and returned to their home cages on 3 consecutive days to habituate them to that cabinet and to minimize the effect of contextual conditioning. On the following 2 days, they were placed in the same chamber to obtain baseline startle values via pre-exposure to 30 acoustic stimuli (noise bursts, 95 dB, 50 ms duration, and 30 s inter-stimulus interval, i.e., ISI). Rats with equivalent baseline mean startle amplitudes were then divided into matched groups. On the day of fear conditioning, each animal was brought to the room, allowed to habituate, and placed in the previously exposed cabinet. The CS-US pairing began after a 5-min acclimation period in this training cabinet.

In the training session, rats in the cabinet received 7 co-terminated light-footshock (CS-US) pairings with an inter-trial interval of 3∼5 minutes. Unpaired controls received the same number of light and footshock presentations, but in a pseudorandom fashion in which the US could occur at anytime except 3.2 s after the CS.

In the testing session, rats were placed in a different cabinet 24 h later, and were pre-exposed to 30 noise bursts (95 dB, 50 ms, 30 s ISI) first. Then they were tested for the fear-potentiated startle, a process involved 10 noise bursts alone (noise-alone trial) and 10 noise bursts presented 3.2 s after the onset of 3.7 s light (light-noise trials). The two trial types were presented in a balanced and mixed order (ISI, 30 s). Potentiated startle (%) was defined as (Startle^light^−Startle^dark^)/Startle^dark^×100

### Cannula Implantation and Intracranial Drug Delivery

Pharmacological intervention experiments were performed before the training session to inspect the two fear conditioned learning pathways, i.e., the BLA→MeA→VMH pathway and the BLA→CeA pathway [15]. Rats were implanted with intracranial cannula 2 weeks before starting the behavioral procedures. These rats were anesthetized as mentioned before and mounted on a stereotaxic instrument with blunt ear bars. The skull was exposed and two stainless steel guide cannulas (23 gauge; Plastics One, Roanoke, VA), which were occluded with an internal dummy stylet extending 1 mm beyond the guide cannula tip, were bilaterally lowered into the brain aiming at the BLA (AP = −2.8 mm, ML = ±5.1 mm, and DV = −8.4 mm), the CeA (AP = −2.6 mm, ML = ±4.2 mm, and DV = −8.5 mm), the MeA (AP = −2.8 mm, ML = ±3.5 mm, and DV = −8.5 mm), or the VMH (AP = −3.2 mm, ML = ±0.7 mm, and DV = −9.4 mm). The cannulas were anchored with dental cement to four jeweler screws that were previously attached to the skull. After one week of resting period to allow recovery from the surgery and acclimation to the startle system, various drugs were bilaterally infused into the rat brain. That is, rats received tetrodotoxin (TTX, Tocris Bioscience, MO; 10 ng in 1 µL to BLA, CeA or MeA), GR 82334 (a SP receptor antagonist from Tocris Bioscience, MO; 6.0 nmol in 1 µL to the VMH), or artificial cerebrospinal fluid (1 µL to the same brain areas). Thirty minutes later, they were placed in the cabinet for the training session of fear-potentiated startle.

### Statistical Analysis

All results are expressed as mean ± SEM in the text and figures. Between-group comparisons were performed using two-tailed Student's *t* tests for independent samples. The pharmacological intervention experiments were analyzed by two-way ANOVA, followed by the Scheffé multiple-range test for post hoc assessment of individual means. For correlation studies, Pearson's correlation analysis (SPSS statistics package, Chicago, IL) was applied. The sample size “n” represented the number of animals in each group. Statistically significant differences were established at p<0.05.

## Results

### Performance of Fear-Potentiated Startle

**Figure 1:**
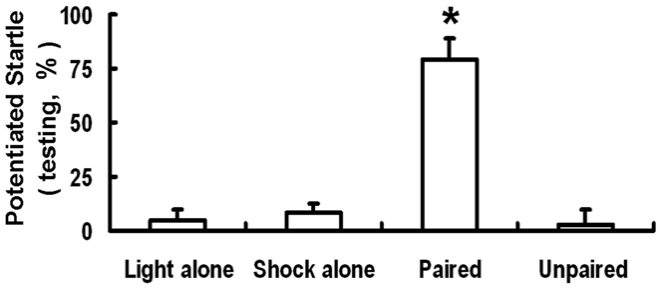
Fear-potentiated startle values in light alone, shock alone, paired CS-US and unpaired CS-US groups. Rats in the paired group received 7 light-shock pairings, whereas the unpaired rats received 7 lights and 7 shocks in a pseudorandom manner. Light alone (n = 8); shock alone (n = 8). * p<0.05, paired (n = 12) vs. unpaired (n = 8).

In order to investigate the learning curve of fear-potentiated startle, we reduced the standard training session from 10∼15 repeated CS-US pairs to only 7 pairs. This shortened training session avoided over-training (saturated learning). Nevertheless our training paradigm was still effective, i.e., the fear-potentiated startle occurred only when the training session consisted of repeated trials of paired CS-US (*t*_(18)_ = 6.34, p<0.05, paired CS-US vs unpaired CS-US, analyzed by unpaired *t*-test) (Fig. 1). The fear-conditioned learning and memory did not take place if the training trials consisted of CS alone, US alone, or unpaired CS-US.

### BP-related Parameters during Learning Trials of the Training Session

**Figure 2:**
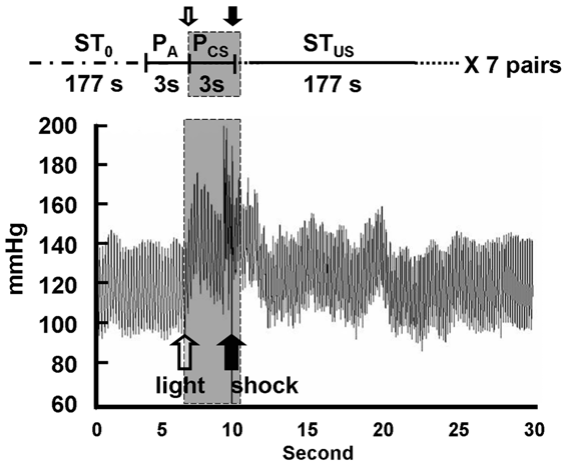
A schematic diagram showing BP alterations induced by light (CS) and footshock (US) during the training session of fear-potentiated startle. The light-on period is marked as the shaded region. P_A_: averaged BP in 3 s before light on; P_CS_: averaged BP in 3 s after light on; ST_0_: averaged resting sympathetic tone in 177 s before the initial P_A_; ST_US_: averaged post-US sympathetic tone in 177 s starting at 3 s after a footshock.

We chose two BP-related parameters, P_CS_/P_A_ and ST_US_/ST_0_, to reflect the physiological responses in the fear-conditioned learning (Fig. 2). First, P_CS_/P_A_ was defined as the post-CS BP (P_CS_; 3-s mean value) divided by the pre-CS BP (P_A_; 3-s mean value) in each trial; it served as an index for the CS-induced rapid elevation of BP. A 3-s interval was used for the calculation because the time interval between CS and US was 3.2 s. Second, relative sympathetic activity (ST_US_/ST_0_) was defined as the post-US sympathetic tone (ST_US_; 177-s mean value) in each trial divided by the “resting” sympathetic tone (ST_0_; 177-s mean value) before the first trial; it served as an index for the US-evoked elevation of sympathetic tone. Data from a 177-s interval was used for calculating the sympathetic tone because i) 3 min was consistently available in all learning trials since the inter-trial intervals were variable (3∼5 min), and ii) data points in the first 3 s right after the US was deleted due to shock-associated noises.

The original BP and sympathetic tone values clearly reflected the physiological responses to the light (CS) and the footshock (US). P_CS_ values (the light-evoked BP levels) were significantly higher than P_A_ values (the pre-light BP levels) under paired CS-US conditions (131.2±4.1 mmHg vs. 118.7±3.3 mmHg, P_CS_ vs. P_A_, *t*_(11)_ = 3.24, p<0.05), but not under unpaired CS-US conditions (125.3±4.3 mmHg vs. 122.6±4.1 mmHg, P_CS_ vs. P_A_, *t*_(7)_ = 0.71, p>0.05, analyzed by paired *t*-test). Additionally, the post-footshock sympathetic activity (ST_US_) values were significantly higher than resting (ST_0_) values under both paired CS-US conditions (65.5±3.7 mmHg^2^ vs. 16.2±1.5 mmHg^2^, ST_US_ vs. ST_0_, *t*_(11)_ = 18.7, p<0.05), and unpaired CS-US conditions (76.1±2.0 mmHg^2^ vs. 16.8±1.4 mmHg^2^, ST_US_ vs. ST_0_, *t*_(7)_ = 80.7, p<0.05, analyzed by paired *t*-test).

**Figure 3:**
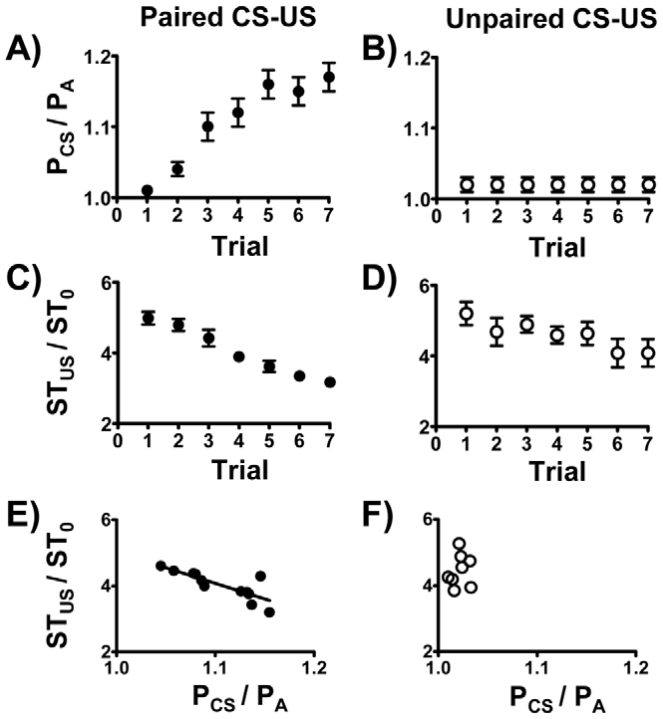
P_CS_/P_A_ and ST_US_/ST_0_ in the learning session. P_CS_/P_A_ represents the CS-induced BP response, while ST_US_/ST_0_ represents the US-induced sympathetic tone response. Both parameters show learning trial-dependent changes under paired CS-US conditions, but not under unpaired CS-US conditions (A–D). Additionally, the trial-averaged values of these two parameters negatively correlate with each other under paired CS-US conditions, but not under unpaired CS-US conditions (E, F). Results were analyzed by Pearson's correlation analysis for individual group.

First, we tested whether these two BP-related parameters (P_CS_/P_A_ and ST_US_/ST_0_) could reflect the steps of fear-potentiated learning and memory. Both parameters served as good indices for fear-conditioned learning responses, as they progressively changed with increasing learning trials under paired CS-US conditions but not under unpaired CS-US conditions (Fig. 3A–D). Interestingly, the learning process was accompanied with increasing P_CS_/P_A_, but with decreasing ST_US_/ST_0_. Additionally, these two parameters negatively correlate with each other under paired CS-US conditions (F_(1,10)_ = 19.38, r = 0.81, p<0.05), but not under unpaired CS-US conditions (F_(1,6)_ = 0.22, r = 0.19, p>0.05) (Fig. 3E, 3F). As a consequence, the trial-averaged values of these two parameters in different animals were negatively correlated to each other under paired CS-US conditions (Fig. 3E). As a comparison, animals subjected to unpaired CS-US conditions showed little BP elevation upon light exposure (P_CS_/P_A_ values were close to 1) and their sympathetic tone remained relatively high throughout the training session (minimal adaptation to repeated shocks).

**Figure 4:**
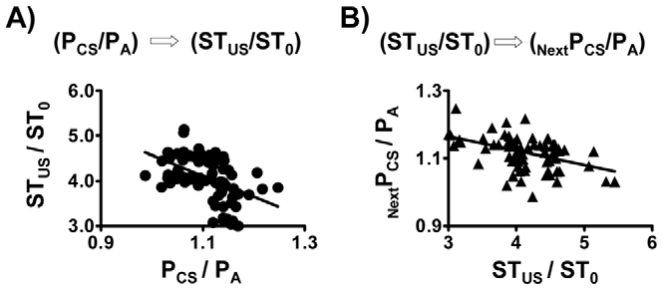
Coupling between P_CS_/P_A_ and ST_US_/ST_0_ in individual trials of the learning session. Data points represent the values of individual trials in the learning session. There were 7 data sets for each animal when P_CS_/P_A_ and ST_US_/ST_0_ values were derived from the same trial, i.e., a CS followed by an US of the same trial (A). There were 6 data sets for each animal when P_CS_/P_A_ and ST_US_/ST_0_ values were derived from two consecutive trials, i.e., an US followed by a CS from the next trial (B). Both parameters correlated with each other either in the same trial or in two consecutive trials.

We then asked whether the negative correlation between P_CS_/P_A_ and ST_US_/ST_0_ in the paired CS-US training session (Fig. 3E) could be due to a two-way coupling between these two parameters. As expected, a high P_CS_/P_A_ value (indicating a good learning score) in a particular trial usually yielded a low ST_US_/ST_0_ of that trial, because the rat was expecting a shock (US) when exposed to the light (CS). There were 7 data sets for each animal when P_CS_/P_A_ and ST_US_/ST_0_ values were derived from the same trial, i.e., a CS followed by an US of the same trial (F_(1,80)_ = 22.27, r = 0.47, p<0.05) (Fig. 4A). There were 6 data sets for each animal when P_CS_/P_A_ and ST_US_/ST_0_ values were derived from two consecutive trials, i.e., an US followed by a CS from the next trial (F_(1,70)_ = 17.13, r = 0.44, p<0.05) (Fig. 4B). A negative correlation was found between P_CS_/P_A_ and ST_US_/ST_0_ when data from 7 learning trials in each animal were plotted together (Fig. 4A). Interestingly, the reverse was also true, i.e., a low sympathetic activity (indicating a relative calm state after the shock) in a particular trial usually yielded a relatively high P_CS_/P_A_ value in the next trial (Fig. 4B). Thus, the post-shock calmness apparently helped identify the light as a cue (CS) later.

**Figure 5:**
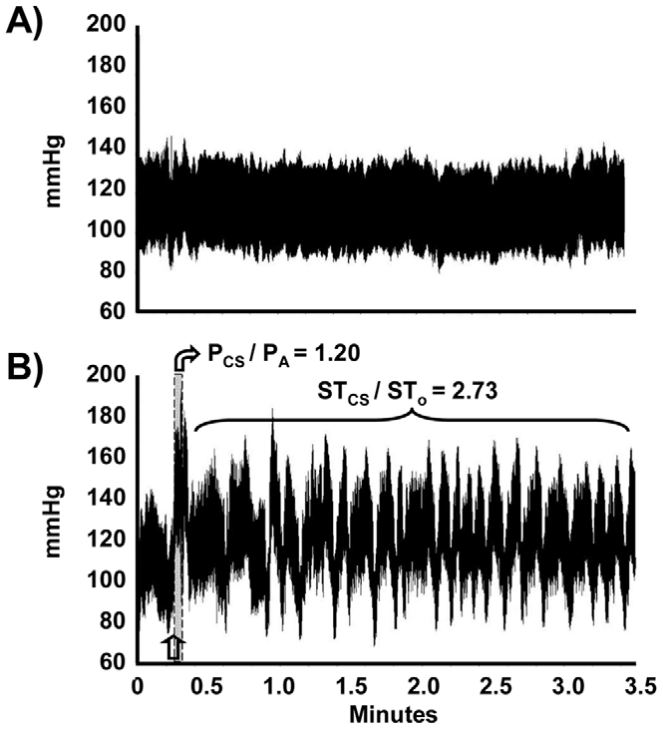
The CS-evoked BP alterations after the completion of a training session with 7 paired CS-US trials. Under our standard protocol, an animal in the training cabinet remained quiescent after finishing the training session (A). As a comparison, if a similarly trained animal encountered an extra CS (B), it showed high values of P_CS_/P_A_ (3-s average) and ST_CS_/ST_0_ (180-s average).

It was intriguing to investigate how trained animals, those that had been repeatedly exposed to CS-US pairs, would behave when encountering an extra CS right after the completion of a standard training session. As expected, the trained animal remained quiescent in the chamber if left undisturbed (Fig. 5A). However, it showed not only an elevated P_CS_ but also a persistently high sympathetic tone (ST_CS_) when exposed to the extra CS (without being followed by an US) (Fig. 5B). The extra CS-evoked P_CS_/P_A_ and ST_CS_/ST_0_ values were 1.20±0.01 and 2.79±0.09, respectively (n = 4). As a comparison, the 7^th^ trial CS-evoked P_CS_/P_A_ values and US-evoked ST_US_/ST_0_ values from the same animals were 1.22±0.02 and 2.73±0.16, respectively (n = 4). While the extra CS did not further increase P_CS_/P_A_ values (1.20±0.01 vs. 1.22±0.02, extra CS trial vs. 7^th^ trial, *t*_(3)_ = 1.61, p>0.05, analyzed by paired *t*-test), it alone was sufficient to evoke ST values (2.79±0.09 vs. 2.73±0.16, _extra_ST_CS_/ST_0_ vs. _7th_ST_US_/ST_0_, *t*_(3)_ = 0.33, p>0.05, analyzed by paired *t*-test). Therefore, trained rats remained relatively uneasy for quite a while even after the light had been turned off at 3.7 s, i.e., they were rather anxious during the “waiting period” for an expected shock.

### BP-related Parameters in the Training Session and the Performance of Fear-Potentiated Startle

**Figure 6:**
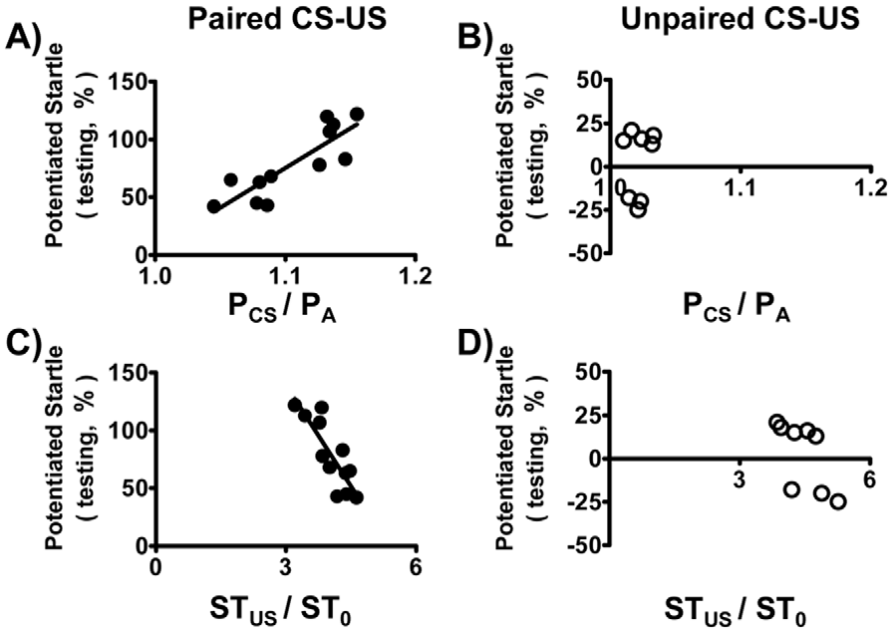
P_CS_/P_A_ and ST_US_/ST_0_ in the learning session forecasted the performance of fear-potentiated startle. The trial-averaged values of both parameters in training sessions under paired CS-US conditions effectively established the fear-potentiated startle (A, C), but those under unpaired CS-US conditions were unable to do so (B, D).

We further tested whether the BP-related parameters in the training sessions could forecast the fear-conditioned memory on the next day. Apparently both parameters faithfully predicted the performance of fear-potentiated startle (Fig. 6). P_CS_/P_A_ values obtained under paired CS-US conditions: F_(1, 10)_ = 26.21, r = 0.85, p<0.05; ST_US_/ST_0_ values obtained under paired CS-US conditions: F_(1, 10)_ = 25.03, r = 0.85, p<0.05 (Fig. 6A, 6C); P_CS_/P_A_ values obtained under unpaired CS-US conditions: F_(1, 6)_ = 0.22, r = 0.19, p>0.05; ST_US_/ST_0_ values obtained under unpaired CS-US conditions: F_(1, 6)_ = 4.41, r = 0.35, p>0.05 (Fig. 6B, 6D). Animals with either high P_CS_/P_A_ values or low ST_US_/ST_0_ values performed well in the task of fear-potentiated startle (Fig. 6A, 6C). As a control, animals subjected to unpaired CS-US training session the previous day showed low P_CS_/P_A_ values and high ST_US_/ST_0_; and they performed poorly in the task of fear-potentiated startle (Fig. 6B, 6D). Please note that P_CS_/P_A_ values under paired CS-US conditions were significantly higher than those under unpaired CS-US conditions (1.106±0.031 vs. 1.022±0.008, paired CS-US vs. unpaired CS-US, *t*_(18)_ = 2.16, p<0.05, analyzed by unpaired *t*-test) (Fig. 6A, 6B). Additionally, the values of ST_US_/ST_0_ under paired CS-US conditions were significantly lower than those under unpaired CS-US conditions (4.03±0.15 vs. 4.71±0.31, paired CS-US vs. unpaired CS-US, *t*_(18)_ = 2.19, p<0.05, analyzed by unpaired *t*-test) (Fig. 6C, 6D).

### P_CS_/P_A_ Values during the Testing Session and the Performance of Fear-Potentiated Startle

**Figure 7:**
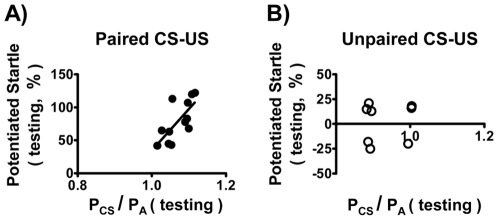
Correlation between potentiated startle and P_CS_/P_A_ (testing) values. P_CS_/P_A_ (testing) values were obtained by measuring the 3-s averaged BP values before and after the light (CS) during the testing session (averaged 10 times of the CS-evoked response for each rat, n = 12).

Strictly speaking, fear-potentiated startle is a unique measure of the Pavlovian fear learning. To test whether P_CS_/P_A_ values during the testing session can reflect the cued fear learning and memory, the correlation between P_CS_/P_A_ (testing) value and fear-potentiated startle was examined. Paired CS-US experiments: potentiated startle vs. P_CS_/P_A_ (testing), F_(1,10)_ = 10.51, r = 0.72, p<0.05 (Fig. 7A); unpaired CS-US experiments: potentiated startle vs. P_CS_/P_A_ (testing), F_(1,6)_ = 0.08, r = 0.11, p>0.05 (Fig. 7B). P_CS_/P_A_ (testing) served as a good parameter for cued response, as its values correlated well with the values of fear-potentiated startle. Moreover, the P_CS_/P_A_ (training) values also faithfully predicted the P_CS_/P_A_ (testing) values on the next day (the cued fear memory, data not shown).

### Pharmacological Interventions to Elucidate Fear-Conditioned Learning Pathways in the Fear-Potentiated Startle

**Figure 8:**
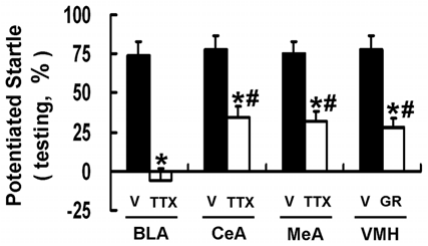
Pharmacological interventions showing two pathways involved in the fear-potentiated startle. The blockers were locally injected into BLA, CeA, MeA or VMH 30 min before the training session to block local neuron activity during fear learning. *p<0.05, blocker-treated vs. vehicle; # p<0.05, other brain areas vs. BLA under blocker-treated conditions. GR: GR 82334 (a SP receptor antagonist), TTX: tetrodotoxin, V: vehicle.

**Figure 9:**
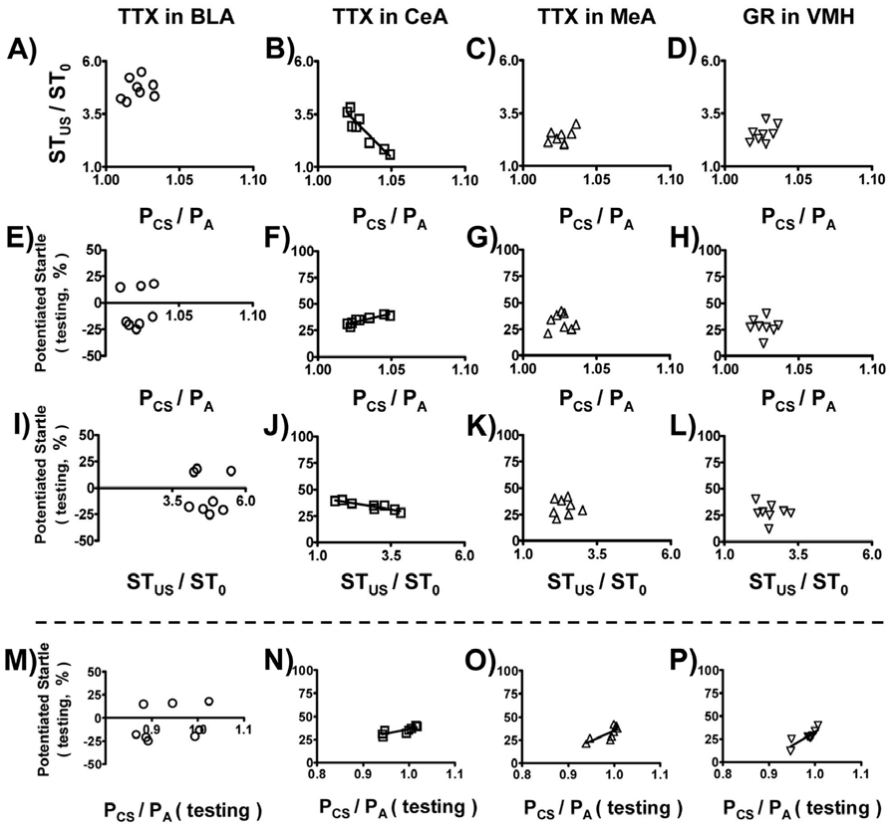
Pharmacological interventions to locally block one or both of the fear learning pathways and the consequences on the fear-potentiated startle. The blockers were locally injected into BLA, CeA, MeA or VMH to block local neuron activity before training session. Results were analyzed by Pearson's correlation analysis for individual group. Panels with line marks indicate significant correlation between the ordinate and the abscissa. Results from measuring P_CS_/P_A_ values in the training session indicated that the MeA/VMH-mediated fear learning pathway, but not the CeA-mediated pathway, was BP-related (A–L). However, local blockages in CeA, MeA, or VMH all retained partial fear learning and memory (M–P).

As mentioned earlier, the conditioned fear learning process involves the activation of BLA which projects to two distinct downstream nuclei in the amygdala, i.e., CeA and MeA. We, therefore, explored these fear-conditioned learning pathways by local injection of neural blockers into BLA, CeA, MeA, and VMH (a downstream component of MeA in the hypothalamus). A two-way ANOVA analysis with treatment (drug vs. vehicle) and brain areas (BLA, CeA, MeA or VMH) as between-subjects factors indicated a significant treatment effect (F_(1,56)_ = 771.61) and also a significant treatment×brain areas interaction (F_(3,56)_ = 19.99). Our results in Figure 8 showed that blocking BLA abolished fear-potentiated startle, while blocking either CeA or MeA/VMH only partially inhibited the performance of this task. These data thus confirmed that two pathways were parallel to each other and that each pathway played a partial role in mediating the performance of fear-potentiated startle. This hypothesis was further supported by measuring our BP-related parameters under local blocking conditions before the training session (Fig. 9). Under paired CS-US conditions, the negative correlation between P_CS_/P_A_ and ST_US_/ST_0_ was abolished when the blockers were locally infused to the BLA, the MeA, or the VMH, but not to the CeA (Fig. 9A–D). Results in Figure 9 top panels: Fig. 9A, F_(1, 6)_ = 0.35, r = 0.23, p>0.05; Fig. 9B, F_(1, 6)_ = 41.59, r = 0.93, p<0.05; Fig. 9C, F_(1, 6)_ = 1.62, r = 0.46, p>0.05; Fig. 9D, F_(1, 6)_ = 1.88, r = 0.49, p>0.05. In parallel, the BP-related parameters were unable to reflect the fear-potentiated startle when blockers were infused to the BLA, the MeA or the VMH; but they remained predictive even when the CeA pathway was blocked (Fig. 9E–L). Results in Figure 9 middle panels: Fig. 9E, F_(1, 6)_ = 0.17, r = 0.16, p>0.05; Fig. 9F, F_(1, 6)_ = 24.19, r = 0.89, p<0.05; Fig. 9G, F_(1, 6)_ = 0.002, r = 0.02, p>0.05; Fig. 9H, F_(1, 6)_ = 0.02, r = 0.06, p>0.05; Fig. 9I, F_(1, 6)_ = 0.005, r = 0.03, p>0.05; Fig. 9J, F_(1, 6)_ = 34.38, r = 0.92, p<0.05; Fig. 9K, F_(1, 6)_ = 0.001, r = 0.01, p>0.05; Fig. 9L, F_(1, 6)_ = 0.26, r = 0.20, p>0.05. Therefore, although the CeA pathway played a partially role in fear-conditioned learning and memory, it did not modulate the BP-related parameters during the training session.

Finally, we asked what the retrieval process of fear memory would be like when one of the two fear-conditioned pathways in the training session was locally blocked. As mentioned earlier, we ascertained that in the testing sessions P_CS_/P_A_ also faithfully reflected the fear-potentiated startle, i.e., a positive correlation between P_CS_/P_A_ (testing) and fear-potentiated startle indicated a successful retrieval of fear memory (Fig. 7). In the testing session, the retrieval process only failed when the BLA (the common initiation site for both pathways) was disabled by tetrodotoxin (TTX) in the training session (Fig. 9M–P). Results in the bottom panels of Figure 9: Fig. 9M, F_(1, 6)_ = 0.50, r = 0.28, p>0.05; Fig. 9N, F_(1, 6)_ = 10.15, r = 0.79, p<0.05; Fig. 9O, F_(1, 6)_ = 8.26, r = 0.76, p<0.05; Fig. 9P, F_(1, 6)_ = 14.81, r = 0.84, p<0.05. Therefore, the retrieval of fear-conditioned learning and memory used both pathways, regardless which pathway was blocked in the training session.

## Discussion

This study is the first to show the significance of monitoring real-time BP changes in rats during training and testing sessions of the fear-potentiated startle. Two BP-related parameters, the CS-induced BP elevation and the US-evoked sympathetic tone elevation (P_CS_/P_A_ and ST_US_/ST_0_), progressively changed with increasing learning trials and they were functionally coupled, indicating two-way interactions between cued learning and post-shock calmness. Besides, both parameters closely predicted the performance of fear-potentiated startle on the next day. Local intracranial blockade experiments confirmed that two parallel pathways were involved in the fear-conditioned learning and memory [6]–[8]; one being BP-related (from the BLA through the MeA and the VMH) and the other being BP-unrelated (from the BLA through the CeA). Regardless either pathway was blocked in the training session for the fear learning, both contributed to the fear memory retrieval.

In principle, an efficient learning should generate a successful outcome in any learning and memory-related task. However, such an argument is often untested because of practical difficulties in quantifying the learning process without using some forms of test, and learning and testing processes frequently interfere with each other. As fear is a form of emotion that often tie together with altered cardiovascular parameters, we thus measured two BP-related parameters during learning trials of the training session and wished them act as real-time indices for fear-conditioned learning. Interestingly, both parameters (P_CS_/P_A_ and ST_US_/ST_0_) not only fulfilled this goal but also coupled to each other, i.e., both progressive changed with increasing learning trials and inversely influenced each other (Figs. 3 and 4), indicating a close relationship between cue identification and post-shock calmness. As a matter of fact, although the “learning curves” for each animal showed occasional up-and-downs, rats showing fast increases in P_CS_/P_A_ also showed rapid decline of ST_US_/ST_0_ (Fig. S1). Judging from the tight correlations between these BP-related parameters and the performance of fear-potentiated startle (Fig. 6), an efficient learning indeed generated a high-quality outcome of the associated memory.

The coupling between CS-evoked responses and US-evoked responses played a pivotal role in fear learning and memory (Figs. 3 and 4). It is intriguing to ask the underlying mechanisms explaining how this kind of coupling was achieved, i.e., how repeated CS-US pairs actually helped animals “learn” the conditioned fear. Stress-activated neurotransmitters and stress hormones enhance the consolidation of memory for emotionally arousing experiences through actions involving the amygdala [18]. However, the glucocorticoid effects on fear memory generally follow an inverted U-shape dose-response relationship; moderate doses enhance memory, whereas high doses are likely to impair memory consolidation [19]. Therefore, animals with suitable amounts of glucocorticoids after exposing to an US would be a good learner, and vice versa. Since rats showed fast increases in P_CS_/P_A_ also showed rapid decline of ST_US_/ST_0_ (Fig. S1), their post-shock glucocorticoid level would be close to the optimal range to enhance fear learning and memory. As for poor learners, they actually showed higher post-shock sympathetic tone than good learners did (Fig. S1). Moreover, the post-shock sympathetic tone was higher in animals treated with unpaired CS-US than those treated with paired CS-US (76.1±2.0 mmHg^2^ vs. 65.5±3.7 mmHg^2^, *t*_(18)_ = 2.19, p<0.05; data taken from Fig. 3). Therefore, the glucocorticoid levels in poor learners for the fear-potentiated startle were most likely too high than too low. Thus treatments capable of reducing the post-shock sympathetic tone would show improved “learning curves” during the training session (repeated exposure to paired CS-US trials).

Employing this real-time monitoring method for obtaining BP and sympathetic tone data in experimental animals, our results provide investigators an opportunity to examine two historically contradictory theories regarding the relationship between emotion formation and physiological responses [20]. Our implanted telemetric probe was capable of not only monitoring animals' physiological responses (BP) but also tracing their emotion-related responses, e.g., the freezing response indicated by lacking of any locomotor activity. This activity-related parameter is semi-quantitative because it is reflected by moment-to-moment changes in the signal strength due to changes in location or orientation of the probe relative to the receiving antenna. Nevertheless, this parameter has a high temporal resolution and can be recorded without interfering with the BP recording. In the testing session indicated, our preliminary results showed that the light-induced freezing (a fear-caused response) seemed to occur earlier than the light-induced BP changes (physiological responses) (Fig. S2). Based on these results, we are inclined to support the notion that the fear emotion formation precedes physiological responses, i.e., physiological responses and emotion formation are likely to be parallel products in response to environmental stimuli rather than physiological responses are a prerequisite of fear memory formation.

As a whole, these two BP-related parameters faithfully quantified the performance of fear learning and revealed the learning progress real-time. This study support negative feedback mechanisms in the amygdala regulating the association formation in Pavlovian fear conditioning [21]. Therefore, these BP-related parameters should not only serve as good indicators for other forms of fear-conditioned learning and memory, but also provide a new way to assess the mechanisms of association formation in the conditioned fear learning and memory (including extinction) [22], [23].

## Supporting Information

Figure S1 **BP-related parameters change in each rat during learning trials of the training session.** The tracings of the P_CS_/P_A_ value are shown in the upper panels (A, B), while the tracing of the ST_US_/ST_0_ are shown in the lower panels (C, D). Although the “learning curves” for each animal in the group of paired CS-US showed occasional up-and-downs, rats showing fast increases in P_CS_/P_A_ (3 best learners marked in blue curves) also showed rapid decline of ST_US_/ST_0_. The vice versa was also true (3 poorest learners marked in red curves).
(TIF)

Figure S2 **The real-time activity-related parameter and BP tracings in the testing session.** This activity-related parameter (MKUs) is semi-quantitative because it basically reflected the moment-to-moment changes in the signal strength (A). Results in the testing session indicated that the light-induced freezing (a fear-derived reflex indicated by lacking of any activity) happened earlier than the light-induced BP changes (a fear-associated physiological response) (B). Shaded area: the duration with light on.
(TIF)
