# Supplementary figures and images for: Blood Pressure Variations Real-Time Reflect the Conditioned Fear Learning and Memory
Source: PLoS One. 2012 Apr 4;7(4):e32855. doi: 10.1371/journal.pone.0032855 (PMC3319555; doi:10.1371/journal.pone.0032855)

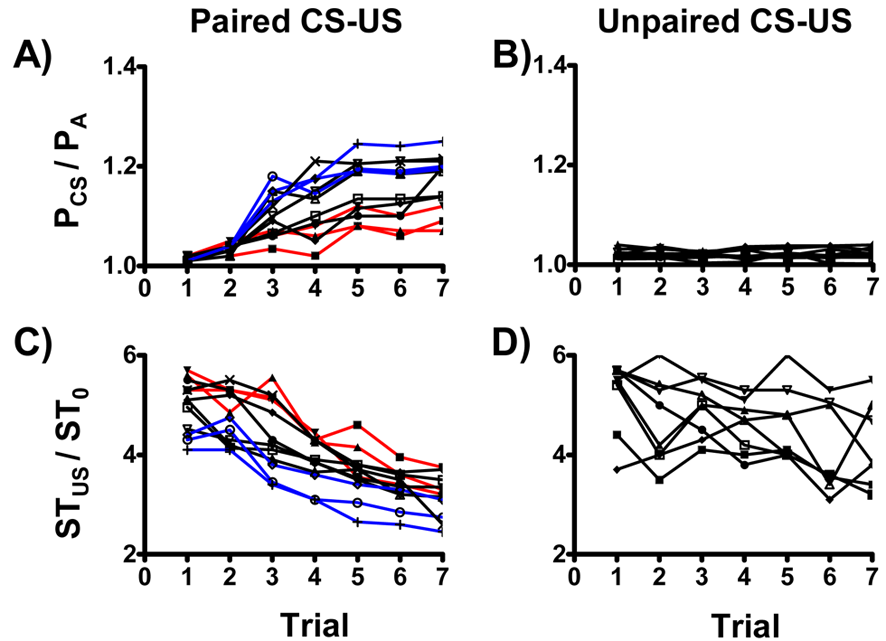

Supplement: Figure S1 — BP-related parameters change in each rat during learning trials of the training session. The tracings of the PCS/PA value are shown in the upper panels (A, B), while the tracing of the STUS/ST0 are shown in the lower panels (C, D). Although the “learning curves” for each animal in the group of paired CS-US showed occasional up-and-downs, rats showing fast increases in PCS/PA (3 best learners marked in blue curves) also showed rapid decline of STUS/ST0. The vice versa was also true (3 poorest learners marked in red curves). (TIF) [file pone.0032855.s001.tif]

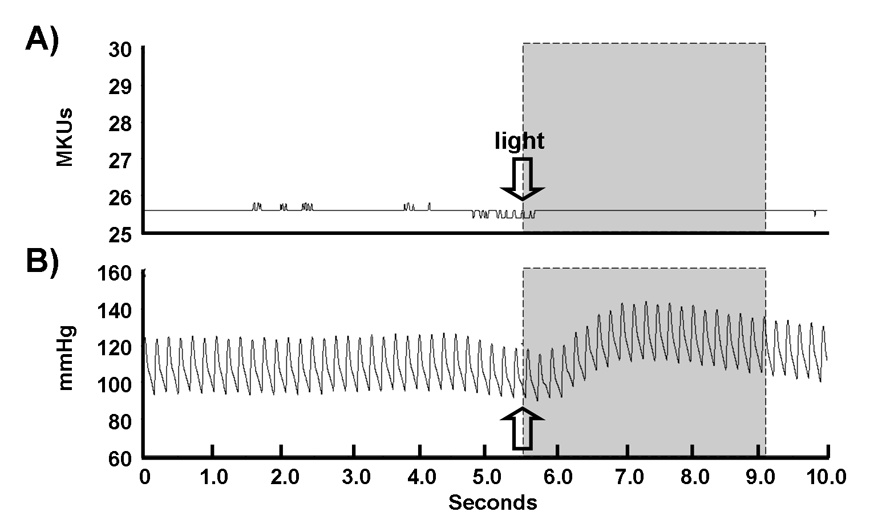

Supplement: Figure S2 — The real-time activity-related parameter and BP tracings in the testing session. This activity-related parameter (MKUs) is semi-quantitative because it basically reflected the moment-to-moment changes in the signal strength (A). Results in the testing session indicated that the light-induced freezing (a fear-derived reflex indicated by lacking of any activity) happened earlier than the light-induced BP changes (a fear-associated physiological response) (B). Shaded area: the duration with light on. (TIF) [file pone.0032855.s002.tif]
